# Role of lysosome in healing neurological disorders by nano-bioengineering

**DOI:** 10.3389/fnins.2023.1331211

**Published:** 2024-01-08

**Authors:** Aiswarya Raj, Urmi Bandyopadhyay

**Affiliations:** Manipal Institute of Regenerative Medicine (MIRM), Bengaluru, Manipal Academy of Higher Education (MAHE), Manipal, Karnataka, India

**Keywords:** lysosome, autophagy, amino acid, nanotechnology, bioengineering, neurodegenerative disorder

## Abstract

Lysosomes primarily recognized as center for cellular ‘garbage-disposing-unit’, which has recently emerged as a crucial regulator of cellular metabolism. This organelle is a well-known vital player in the pathology including neurodegenerative disorders. In pathological context, removal of intracellular damaged misfolded proteins, organelles and aggregates are ensured by ‘Autophagy’ pathway, which initially recognizes, engulfs and seals the toxic cargo at the cytosolic environment. Thereafter the cell completes the task of encapsulated cargo elimination upon delivery of them to the terminal compartment - lysosome, which contains acid hydrolases, that are capable of degrading the abnormal protein-lipid-repertoire. The merge between inseparable ‘Autophagy’ and ‘Lysosomal’ pathways evolved into ‘Autophagy-Lysosome Pathway (ALP)’, through which cell ultimately degrades and recycles bio-materials for metabolic needs. Dysregulation of any of the steps of the multi-step ALP can contribute to the development and progression of disorders including Alzheimer’s disease (AD), Parkinson’s disease (PD), and Huntington’s disease (HD). Therefore, targeting differential steps of ALP or directly lysosomes using nano-bioengineering approaches holds great promise for therapeutic interventions. This review aims to explore the role of distal autophagy pathway and proximal lysosomal function, as cellular degradative and metabolic hubs, in healing neurological disorders and highlights the contributions of nano-bioengineering in this field. Despite multiple challenges, this review underscores the immense potential of integrating autophagy-lysosomal biology with nano-bioengineering to revolutionize the field and provide novel therapeutic avenues for tackling neurological-neurodegenerative-disorders.

## Introduction

### Historical perspective and summary

Cellular health relies on maintenance of proper cellular-metabolic function, which is heavily dependent on clearance of damaged cellular materials. Lysosomes are the key organelle for cellular protein degradation and clearance. Malfunction of lysosome is one of the major causes for multiple degenerative-disorders, including, neurodegeneration, which manifests the importance of this organelle in pathophysiological contexts (Wong and Cuervo, 2010). The cluster of neurological disorders pose significant challenge in healthcare, requiring special clinical attention in combatting these complex multi-faceted diseases. In this regard lysosomes can play pivotal role in designing innovative therapeutic-strategies against neurodegenerative disorders, as lysosome is recently conferred with multi-modal role in cellular-metabolism. It is important to highlight that, apart from its conventional role in ‘garbage-recycling’, the newly-emerging concept is further establishing lysosome as the major signaling-hub for cellular metabolite storage and homeostasis, which is vital for sensing and maintaining the intracellular-extracellular metabolite balance. However, relevance of all these aspects in the context of neurodegeneration and neurotrauma is a mysterious black-box. The primary feature of this review is to explore the avenues to therapeutically target the multi-dimensional role of autophagy-pathways and lysosomes in catabolism and in conventional and/or unconventional metabolism, using newly-developed ‘Nanotechniques and Bioengineering’ approaches, to better tackle the various neurodegenerative disorders.

**Figure 1:**
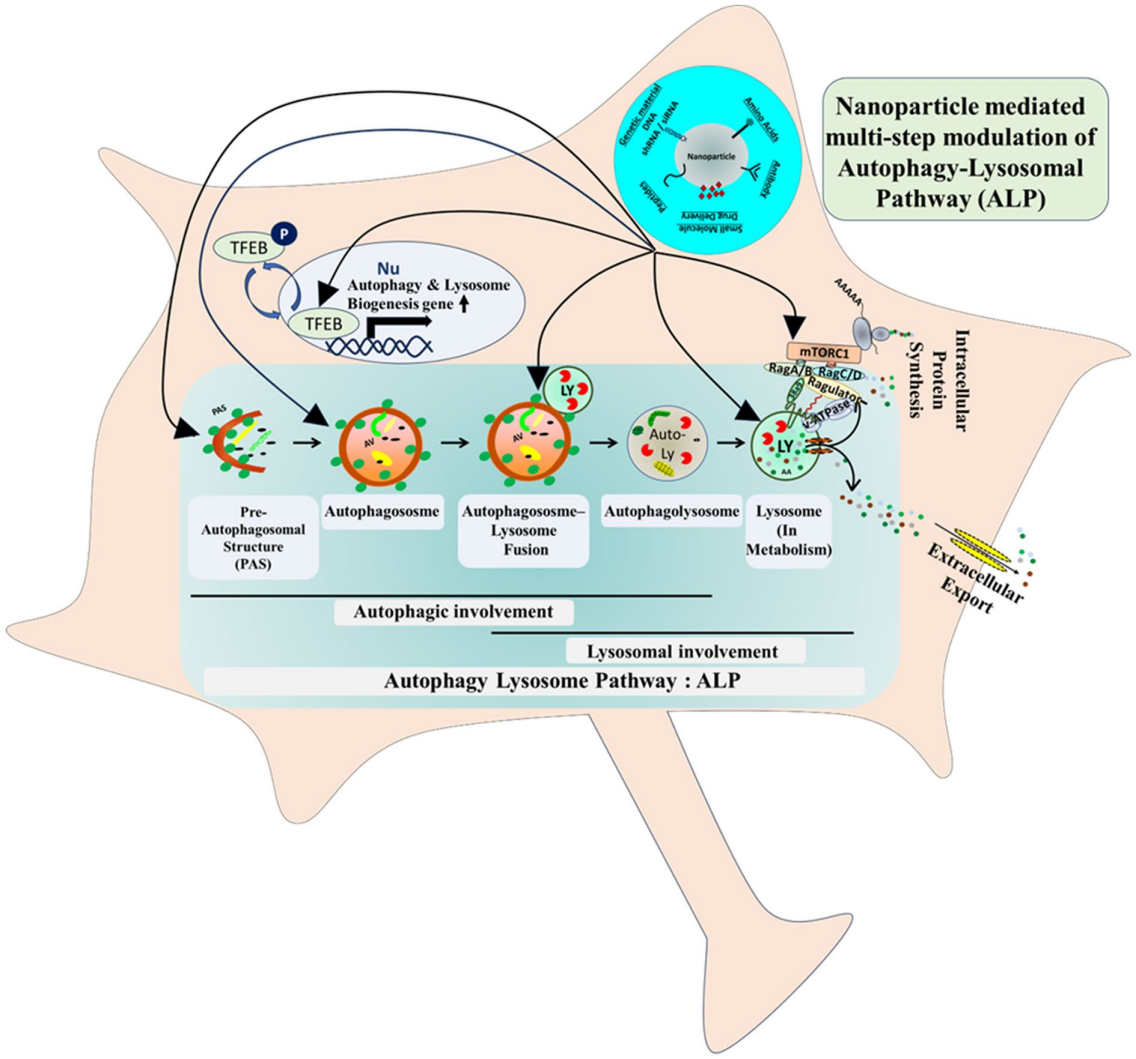
Nanoparticle (NP) mediated multi-step modulation of autophagy-lysosomal pathway (ALP).

The hallmark of neurodegeneration is cellular and lysosomal aggregate formation. There are contradictory concepts prevailing in the neurobiology field, supporting the advantages and disadvantages of aggregate formation in neurodegeneration. No matter whatever the cause and effect of the aggregation process is, subsequent shielding and removal of the aggregated-proteins have been shown to be beneficial even at the clinical level (Karran and De Strooper, 2022). So far, the aggregate removal process is tightly inter-linked with the autophagy pathway, more precisely bulk-degradative macroautophagy pathway, where aggregates and damaged proteins are being sequestered by pre-autophagosomal structures (PAS/phagophore), which upon sealing (autophagosome formation) delivers the luminal cargo to lysosomes for degradation in a fusion dependent manner (He and Klionsky, 2009; Yamamoto et al., 2023) (Figure 1). In brief, the ALP is a complex multi-step pathway. At the distal-end or upstream of it, initial PAS formation occurs followed by engulfment of cytosolic protein-aggregate/abnormal-protein/organelle (cargo). This is then followed by sealing of the double-membraned autophagosomal structures that completes the autophagosome/autophagic-vacuole formation. At the proximal-end or downstream of it, this cargo-carrying autophagosomes then delivers their cargo to lysosomes upon fusion, that ultimately generates autophagolysosomes/autolysosome. Finally, within the autolysosome the entrapped materials get degraded by variety of lysosome-residing acid-hydrolases. With the advent of the field of nano-biotechnology, alteration of this multi-step, highly complex, fine-tuned clearance pathway using bioengineering-nanotechnology could potentially be an effective strategy to better tackle progressive neurodegenerative disorder (Figure 1).

On the other hand, ours and other groups’ research have shown lysosomal-metabolite-storage in response to altered nutrient composition in cellular-niche (Abu-Remaileh et al., 2017; Bandyopadhyay et al., 2022). This lysosomal metabolite-storage-reservoir has been shown to be utilized for cellular protein synthesis that decides the cell fate - growth, proliferation and survival. In contrast to the proliferating cells, lysosomal metabolite storage capacity is compromised in senescent cells. These suggest possibility of varying cellular metabolite homeostasis, requirements and utilization-pattern for cellular growth, proliferation and survival in different cell sub-types within a cell-populations. This could be far more relevant in neurodegenerative disorders, where mixed neuronal cell-population with varying abnormalities exists in a particular brain-niche. Further the significance of the niche-nutrient composition in nurturing the metabolically diverse cell-population is highlighted from the recent clinical trials in neurodegeneration. Recently, in case of the most devastating neurodegenerative disorder, AD (tauopathy), alteration of amino acid (AA) levels in patient diet has turned out to be a clinically effective strategy (Sato et al., 2021). However, variation of metabolic state at the individual cell level within a mixed neuronal cell-population, could render the wholistic niche-metabolite alteration less effective and could even be detrimental. Therefore, between the affected versus unaffected neuronal population in neurodegeneration, local concentration of AAs/metabolites are needed to be altered, leaving the surrounding unperturbed. In that case, ultimately adjustment of local, targeted nutrient-level will be therapeutically far more effective. Moreover, the global change in nutrients in the microenvironment can eventually alter mTOR-dependent as well as mTOR-independent AA-homeostasis-pathways in cell (Abu-Remaileh et al., 2017; Bandyopadhyay et al., 2022). This will ultimately lead to unwanted alteration of lysosome-autophagy-pathways at the molecular level, which could have disastrous effect on healthy versus affected neuronal populations. These poorly effective universal flooding-strategy should be essentially replaced with the restricted targeted delivery approach to particular cell-populations, using bioengineered-nanoparticles carrying selective set of biomolecules, e.g., bioengineered AAs/metabolites/drugs/genetic-modifiers (siRNA/shRNA gene silencing tools).

Specifically, targeting the newly-emerging signaling hub – lysosome and related ALP to curb neurodegeneration with the latest bioengineering-nanotechnologies, could potentially be an efficient strategy from therapeutic standpoint and is being discussed in this review.

## Current state of the art

### Tackling neurodegeneration with bioengineering-nanotechnologies

The trademark of the neurodegenerative disorders is the formation of small/large aggregates at the cellular level, in the cytosol as well as within the lysosomes of the diseased-neurons, that are residing within the heterogeneous cluster of healthy and abnormal neurons. Therefore, therapeutically early-detection of abnormality in a targeted manner within particular brain-region or in particular set of neurons, will facilitate the treatment process manifold. Recently, the advent of theranostics dual-strategic approach, unraveled new hope for simultaneous targeting of affected cell detection as well as the localized treatment options for the diseased neuronal-population. This strategy has already been successfully applied for detecting amyloid-beta (Aβ) plaques at the initial stage in AD-patient brain-neurons, just by delivering magnetic resonance imaging (MRI)-competent anti-Aβ antibody/peptide/probe-functionalized nanoparticles (NPs), coupled with simultaneous treatment-effective drug-targeting to the diseased-neurons, using bioengineered-nanoparticles having blood brain barrier (BBB)-crossing ability (Moorthy and Govindaraju, 2021). From this kind of study, it can be easily appreciated that for successful, targeted localized-drug-delivery, accuracy on the defect detection front is vital. Moreover, as early detection can lead to better cure probability, many researchers have been focusing at the detection end of the broad spectrum of neurological disorders treatment. Conceptually, all these precise aggregate detection processes, including antibody-conjugated-nanoparticles, peptide/probe-functionalized nanoparticles, nanobodies, nanocrystals, liposomes, polymer micelle, gold nanoparticles, dendrimer, iron-oxide nanoparticle, carbon nanotubes, will not only detect the aggregates but also simultaneously isolate the undigested-toxic materials in cytosol. This can prepare them for their bulk-engulfment and further degradation by ALP in cell. In brief, mechanistically the antibody-coated aggregates can be recognized as intracellular substrate and ultimately being encapsulated in cytosol by autophagosome, which will subsequently be delivered to lysosome for further degradation and removal.

In that scenario, in order to ensure a successful cellular aggregate removal, one can imagine alteration of multiple steps of autophagy-lysosomal-machinery - either by targeting autophagy machinery upstream or lysosomal-activity downstream. Therefore, the latest bioengineering-nanotechnological approaches, will turn out to be an effective strategy from clinical angle (Figure 1).

### Lysosome-autophagy pathway modulation: targeting autophagy pathway

As discussed earlier, the distal-end of the lysosome-autophagy pathway – the initiation of autophagosome, PAS/phagophore formation, cargo-entrapment and sealing-processes, can be modulated and already proven to be beneficial in case of multiple neurodegenerative disorders. With the advent of the field of nano-biotechnology, alteration of this multi-step, highly complex, fine-tuned clearance pathway using nanotechnology could potentially be an effective strategy to better tackle progressive neurodegenerative disorders (Figure 1). In Table 1 multiple precedence of targeting autophagy pathway at different steps, via newly-emerging bioengineered-nanotechnological tools, in neurological-disorders and in brain-cancer (e.g., neuroglioma and glioblastoma), has been illustrated.

**Table 1:** Autophagy pathway modulation using nano-bioengineering.

Material delivered/type of nanoparticle (NP)	Composition of nanoparticle (NP)	Cargo delivered/function and disease application	Ref.
Drug and protein delivery via miscellaneous NP	Hyaluronic Acid (HA), the negatively charged hydrophilic linear glycosaminoglycan coated PLGA (poly (lactic-co-glycolic acid)) core-shelled NPs	Co-delivering chloroquine, the lysosome alkalizing agent. Lysosome-mediated death of cancer cell and degenerated neurons (extrapolation).	Paskeh et al. (2022)
	Alginate, starch, the natural polymeric modifications on NPs	Delivering autophagy regulators in cancer cells and degenerated neurons in neurodegeneration (extrapolation).	Paskeh et al. (2022)
	Rapamycin-loaded folate-modified liposomes	Delivering Rapamycin (potent mTOR inhibitor) induces autophagy in cell, providing better BBB-crossing-ability.	Paskeh et al. (2022) and Yoon et al. (2019)
	Hydroxychloroquine (HCQ) carrying peptide-modified liposomes	HCQ facilitates increased lysosomal-pH induced local autophagy inhibition, and provides better BBB crossing ability.	Paskeh et al. (2022)
	Polymeric and Cu (I)-catalyzed Micelle loaded with autophagy inhibitors wortmannin and chloroquine	Wortmannin inhibits autophagy induction (PI3K inhibitor) and chloroquine de-acidifies lysosome, both have shown effectiveness in diseases.	Paskeh et al. (2022)
	Cerium oxide NP, NP with TPP-conjugated cerium oxide, Quantum dot NP, Metal Ultrathin gC3N4 NP, Mesoporous Metal ion silica NP, Single-wall carbon nanotube	They have been successfully tested in glial cells from AD mouse model (TgCRND8) to improve defective ALP to maintain normal neuronal hemostasis.	Hajipour et al. (2017)
	Beclin1 and LY294002 (LY) drug encapsulated hydrophilic polymeric NPs	Beclin1, autophagy activating protein and LY drug, autophagy (PI3K) inhibitor for upregulating and downregulating of ALP in cancer therapy and neurodegenerative disorders (predicted).	Paskeh et al. (2022) and Bento et al. (2016)
Metallic NPs	Gold(I) compounds (Au(I)) encapsulated, pH-sensitive self-assembling, micelle-type poly (β-amino ester) containing NPs	NP delivery to lysosome leading to Autophagy induced cell death.	Paskeh et al. (2022) and Lin et al. (2015)
	Titania-coated Gold (Au) nano-bipyramids	Suppressing brain cancer by preventing ALP by inhibiting autophagosome-lysosome fusion in cell. This strategy can be extrapolated for treatment of neurodegeneration.	Paskeh et al. (2022)
	Gold NPs coated with βCas (beta-casein) (Intracardial administration)	Mitigates the toxicity and rescuing AD-like symptoms of Aβ injected zebrafish model system *in vivo*.	Javed et al. (2019)
	Silver (Ag) and Iron (Fe) NPs	ROS over-production triggered cancer cell apoptosis through autophagy over-activation. Applicable for defective neuron elimination in neurodegeneration.	Paskeh et al. (2022)
Miscellaneous Nano-materials	Carbon-based NPs and graphene nanomaterials	Functions in similar manner as above	Paskeh et al. (2022)
Drugs and Genetic tools delivery	‘Green-modified Nanoparticles’: Designed using biocompatible polymers derived from natural sources with high cargo delivery efficiency	Used for siRNA and shRNA delivery to target multiple steps of ALP in degenerative disorders.	Paskeh et al. (2022), Ates et al. (2020), and Bastaki et al. (2021)
	Liposomes based nanocarriers (Green-modified NPs)	In similar manner as above, used for genetic material and drug delivery *in vitro* and *in vivo*.	Paskeh et al. (2022)
	Micelle based nanocarriers (Green-modified NPs)	Functions in similar manner as above.	Paskeh et al. (2022)
	Chitin-derived chitosan (CS)-based nanocarrier (Green-modified NPs)	Targeted delivery of Atg5 (key ALP component) shRNA suppresses autophagy pathway in lung cancer cells. This can be extrapolated to neurodegeneration.	Zheng et al. (2017)
	Polymeric and Cu (I)-catalyzed Micelle loaded siRNA (Green-modified NPs)	Used for ULK1 (ALP initiator) siRNA delivery. This can be extrapolated to neurodegeneration treatments.	Paskeh et al. (2022)

Additionally, neuroprotective nano-biological treatment avenues with rapamycin-loaded liposome induced inhibition of master AA homeostatic regulator - mTOR signaling pathway could potentially be effective (Table 1). The reciprocal relationship between mTOR and autophagy in this scenario could indirectly lead to ALP activation in neuronal system, which in turn can facilitate aggregate removal from diseased neuronal-population. This vital nanotechnology-based modulatory way of mTOR-autophagy-lysosome pathway, could potentially turn out to be effective potential therapeutic intervention and may open up new avenues for neurodegenerative disorder treatment by exploring the cellular metabolism and degradative juncture in future (Maiese, 2016).

### Lysosome-autophagy pathway modulation: targeting directly the organelle - lysosome

So far, the autophagy pathway modulation with nanotechnological advances has been highlighted. However, it is needless to emphasize that the downstream part or the end-point of ALP, majorly the lysosomal compartment, is of prime importance. Therefore, change in lysosome directly through nanotechnological devices, could be advantageous for arresting degenerative disorders. Recent research in AD field, uncovered effectiveness of immunotherapy-driven aggregate removal conceivably by lysosome (Karran and De Strooper, 2022). This emphasizes the importance of boosting the compromised lysosomal degradative-efficiency with the help of bioengineered-nanotechnological tools. Ideally, this can be achieved by generating bioengineered super-functional lysosomes by nanoparticle-mediated direct delivery of activated lysosome-residing enzymes, including lysosomal acid-hydrolases (proteases-cathepsins/phosphatases/nucleases/glycosidases/peptidases/sulphatases/lipases), lysosomal-enzyme activators, metabolites, AAs, peptides, proteins, directly to the lysosomal compartments, which might have higher ability to dissolve cellular-lysosomal aggregates. As a treatment measure of Covid-19, inhibition of lysosomal luminal proteases by endocytic entry and subsequent lysosomal delivery of negatively surface-charged mefloquine loaded poly-(glycerol monostearate-co-ε-caprolactone) nanoparticles (MFQ-NPs) to lungs-cells, have been shown to mitigate SARS-CoV2 infection (Petcherski et al., 2023). Identical nanoparticle-mediated intervention can be applied to alter lysosomal-activity in the context of neurodegeneration.

Although, impaired lysosomal activity/degradation due to lysosomal acidification-loss in multiple familial neurodegenerative disorders is detrimental (Colacurcio and Nixon, 2016), this can be hypothetically utilized for therapeutic advantage upon combining with the alkalinizing MFQ-NP-delivery strategy. Here, the lysosomal de-acidification can be further aggravated in neurodegeneration. This in one hand can be harmful for neurons, whereas counterintuitively can trigger pathogenic aggregate excretion and clearance from the defective neurons by lysosomal exocytosis, due to their complete loss of protein-degradation-ability of modified lysosomes in extreme pH-escalation. In fact, lysosomal exocytosis-mediated aggregate removal strategy has already been proven to be effective in synucleinopathy model system (Xie et al., 2022). Essentially the deleterious effect of lysosomal pH-hike is counterbalanced by its over-induction, which in turn lead to better neuronal health.

Additionally, it is worth highlighting couple more ALP-components, that have been showing significant promise in the field of nanoparticle-mediated therapeutics.

- Upstream of ALP, TFEB (transcription factor EB), the master transcriptional controller of lysosomal-biogenesis and autophagy-induction, plays a vital role in multiple neurodegenerative disorders. At the molecular level, phosphorylation-dephosphorylation-cycle dependent cytosol-nuclear shuttling of TFEB regulates its activity, therefore ALP-activity, mTORC1-kinase-activity and hence overall cellular metabolism (Figure 1). Given the pivotal roles of TFEB, researchers have considered TFEB-level manipulations, using Ceria-nanoparticles. Cerium oxide nanoparticles, which has been shown to be activating TFEB and ultimately promotes the clearance of toxic aggregates in diseased cells (Song et al., 2014). Moreover, the anti-oxidant property of this nanoparticle can potentially mitigate the ROS-related-toxicity in defective-neurons, which is the common feature of affected neurons in neurodegenerative disorders.
- Currently histone deacetylase (HDAC), as a TFEB-deacetylating component became a lucrative target for ongoing clinical trial for HD, ALS and SMA (spinal-muscular-atrophy), where nanoparticle-mediated delivery of HDAC-inhibitors/siRNAs was successfully able to alter ALP *in-vivo* (Li et al., 2022).

Overall, this kind of multi-modal approaches would provide great promise in treating brain-disorders, where proper restoration of dysregulated autophagy, lysosomal activity and biogenesis will potentially be fruitful.

### Targeting lysosomes in a *‘Macrophagic Way’* using nano-bioengineering

Recently nano-bioengineered macrophage in therapeutically tackling bacterial-infections in a transfer-based approach shows great clinical impact, which can be extrapolated to the neurodegeneration field. Directed delivery of drugs/proteinaceous/genetic-materials to the endolysosomal-systems or to the affected cell population niche can be programmed by exploiting the over-active engulfment-endocytotic-exocytotic property of macrophages, which are triggered by immuno-inflammation during infection, injury and neurodegeneration (Huang et al., 2022; Wang et al., 2023). In support, recently intravenous administration of macrophages carrying particular gene-modifying nanoparticles within their endolysosomal compartments has been shown to be efficiently targeted to the PD-affected inflamed mouse brain-regions to ensure local delivery (Haney et al., 2020). Further the elevated engulfment-export strategy of macrophages can be utilized therapeutically for futuristic AA/metabolite-filled UV irradiated apoptotic corpse delivery to the affected neuronal-niche in neurodegeneration (Weavers et al., 2016). Perhaps this could preferentially alter niche-AA and cellular-metabolism in neurodegenerative disorders. Alternatively, macrophages carrying nanoparticles filled with AA/metabolite-cocktail can be targeted directly to the site of disorder, which can ensure direct transfer of nanoparticle-conjugated-AA/metabolite from carrier macrophage (donor) to the recipient neighboring diseased neurons (acceptor). Targeted delivery of peptides/hormones/drugs/genetic-materials (siRNA/shRNA) can be executed in similar manner to regulate metabolism in neurodegeneration.

### Regulation of cellular-lysosomal metabolism by targeted AA/metabolite delivery using nanotechnology-bioengineering in disease

As the undeniable importance of lysosomal function and metabolism in neurodegeneration is beginning to emerge, further discussion is needed to find out the role of nano-bioengineering in cellular-lysosomal metabolism in neurodegeneration. It can be speculated that damaged protein aggregation can alter neuronal environment and ultimately manifest metabolic abnormality in neurodegeneration. In support, niche-guided compromised lysosomal metabolite-storage and related aberrant protein synthesis in senescent cells, mimicking post-mitotic neurons, have been noticed (unpublished data). Moreover, the impact of extracellular-AAs/metabolites in controlling intra-cellular metabolism is supported by multiple clinical trials in neurodegeneration. Recently, researchers have, (i) identified symptom-relieving effect of dietary administration of selective-essential-AAs in aging and tauopathy mouse-models (Sato et al., 2021); (ii) uncovered AD-symptom manifestation in patients is tightly coupled to lower blood levels of essential-AAs/Brunched-Chain-AAs (BCAAs) in a cohort study; (iii) identified effective reversal of ASD (Autism-spectrum-Disorder) behavior-symptoms upon dietary interventions with AA-cocktails in patients and mice. At the molecular level this could be an effect of mTOR-signaling suppression by autophagic upregulation (Wu et al., 2017; van Sadelhoff et al., 2019). Based on these studies, the promising strategy of external-AA/metabolite level modulation could be applied for treatment of neurodegenerative disorders. However, the wholistic AA/metabolite concentration change as well as global delivery of proteins and genetic materials (including, plasmids/DNA/shRNA/siRNA/miRNA; Siafaka et al., 2022; Duan et al., 2023), within a mixed neuronal-population could have limited efficacy, as region-specificity has been evidenced in patient-brains in neurodegeneration. For example, HD affects the basal ganglia region, whereas hippocampal, entorhinal and cerebral cortical zone is affected in AD, substantia nigra is affected in PD and set of brain-spinal cord motor neurons are the vulnerable population in ALS. This emphasizes the importance of targeted-delivery-mode in order to better control this cluster of disease.

As discussed earlier, uniform spreading-strategy of AAs/metabolites could even be detrimental where local alteration is preferred. At the molecular level, the global, non-targeted, uncontrolled change in the nutrients in the microenvironment can eventually alter mTOR-dependent as well as mTOR-independent AA-homeostasis that can lead to aberrant ALP alteration causing neuronal health deterioration within a population. Therefore, it is essential to replace the existing global AA/metabolite-treatment-strategy with futuristic restricted, targeted delivery-approach to the particular cell populations in a region-specific manner. In this scenario, contribution from the Bioengineering-Nanotherapeutic-technological advances will be extremely beneficial for targeted delivery of selective set of AAs/proteinoids/lysosomal-activity-modifying-agents, through nanoparticle-based carriers. The bi-modal effectiveness of nanotechnology-based carriers is undeniable, as not only they are ensuring targeted delivery to specific neuronal populations in particular affected brain regions, but also capable of protecting the molecular integrity of the modifiable and degradation-prone proteinaceous and genetic-materials. To this end, examples of nanoparticles are highlighted in Table 2, which can either carry protein/peptide/genetic-materials/drugs in an encapsulated or surface conjugated manner (Kang et al., 2018; Siafaka et al., 2022; Duan et al., 2023), to facilitate BBB-crossing to initiate localized treatment for neurodegeneration.

**Table 2:** Nanoparticles in neurodegenerative disorder.

Mode of action	Disease treated	Type of nanoparticle	Functionality and materials delivered	Ref.
Detection of neurodegeneration	AD	Polyethylene glycol (PEG)–polylactic acid (PLA) coated SPION: Super Paramagnetic Iron Oxide Nanoparticle	Successfully detects Aβ-plaques in AD upon penetrating BBB	Hajipour et al. (2017) and Cheng et al. (2015)
		Anti-Aβ antibody functionalized/composed Super Paramagnetic Iron Oxide Nanoparticle (SPION)s	Possesses BBB crossing ability and effective amyloid plaques detection ability	Hajipour et al. (2017) and Sillerud et al. (2013)
		Immunomagnetic Nano-Bio-sensor – Anti-Aβ antibody (AD-marker) associated with N-graphene	Successfully used for Aβ-plaques detection	Hajipour et al. (2017) and Sillerud et al. (2013)
		Hybrid graphene oxide NP with magnetic/plasmonic core	Selectively identifies Tau, AD-biomarkers in blood of AD-patients	Hajipour et al. (2017) and Demeritte et al. (2015)
		Conjugated gold Nano-Particles with Tau antibodies	This NP has been used for Tau protein detection in cerebrospinal fluid (CSF)	Hajipour et al. (2017) and Neely et al. (2009)
		Magnetic Nano-Particles with sialic acid-containing glycosphingolipids (ganglioside)	Exhibiting high efficacy in targeting and strong affinity for Aβ that ease the Aβ early detection process	Hajipour et al. (2017) and Kouyoumdjian et al. (2013)
Cargo delivery (biomolecules/genetic materials)	AD	Exosomes	In AD-pathology engineered exosome deliverying BACE1 (Beta-secretase 1) siRNA in mouse brain cortical region.	Alvarez-Erviti et al. (2011)
		PLGA nanoparticles: poly(D, L-lactic acid-co-glycolic acid) nanoparticles	Used *in vivo* in AD-pathology for targeted delivery of proteins, peptides, genetic material and growth factors.	Siafaka et al. (2022)
		Nanoparticles with lecithin modification with polyethylene glycol-polylactide-polyglycolide (PEG-PLGA)	Similar usage as above	Siafaka et al. (2022)
		Crosslinked starch nanoparticles	Similar usage as above	Siafaka et al. (2022)
		Pegylated PLGA nanoparticles	Similar usage as above	Siafaka et al. (2022)
		Pegylated PLA nanoparticles	Similar usage as above	Siafaka et al. (2022)
		poly ethylene glycol nanoparticles	Similar usage as above	Siafaka et al. (2022)
		PEGylated dendrigraft poly-l-lysins (DGLs) nanoparticles	Similar usage as above	Siafaka et al. (2022)
	PD	Exosomes	Nano scavenger delivering genetic materials and drugs in α-synuclein aggregate clearance, cytotoxicity reduction in neurons in PD mice.	Liu et al. (2020)
		Poly (butly cyanoacrylate) (PBCA) nanoparticles	This NP ensures delivery of Nerve growth factors (NGFs), acteoside, pDNA, Rabies virus glycoprotein (RVG) peptide, miRNA-124 and siRNA	Siafaka et al. (2022)
		Chitosan PEG–PLA nanoparticle	Similar usage as above in PD	Siafaka et al. (2022)
		Exosome curcumin/phenoboronic acid-poly(2-(diethylamino)ethyl acrylate) nanoparticle	Similar usage as above in PD	Siafaka et al. (2022)
		Polymeric nanoparticles	Similar usage as above in PD	Siafaka et al. (2022)
		Pegylated PLA	Similar usage as above in PD	Siafaka et al. (2022)
		PLGA nanoparticles	Similar usage as above in PD	Siafaka et al. (2022)
		Polyethyleneimine nanoparticles	Similar usage as above in PD	Siafaka et al. (2022)
	HD	PLGA	Cholesterol and siRNA delivery	Siafaka et al. (2022)
		Chitosan nanoparticles	Used for biomolecules in HD	Siafaka et al. (2022)
Genetic Material Delivery	AD, PD, HD	Exosome	Targeted delivery of therapeutically active molecules to specific mouse brain cell types and in patients with AD, PD and HD.	Alvarez-Erviti et al. (2011), Didiot et al. (2016), Izco et al. (2019), Zhai et al. (2021), and Jahangard et al. (2020)
		Cx43/L/CS (Connexin 43/ lipid bilayers/ chitosan)-siRNA-RVG peptide nanoparticles	Similar usage as above	Lu et al. (2019)
	Multiple neurodegenerative disorders	Manganese-containing chitosan-matrix nanoparticles (mNPs)	Non-invasive nano-therapeutic siRNA or dsDNA delivery showing promising effect on mouse brain-regions *in vivo* and could be extrapolated to patients with brain disorders.	Sanchez-Ramos et al. (2018)
Others		Lipid-based, inorganic inert Nano systems, including gold-nanoparticle and nanorod	Targeting diseased neurons in neurodegeneration	Siafaka et al. (2022)

Altogether, all the above-mentioned nano-bioengineering strategies could be applied to alter autophagy-lysosome regulated cellular metabolism in disease context. Either genetic alteration of candidates of canonical mTORC1-pathway (Abu-Remaileh et al., 2017) and/or noncanonical lysosomal-AA-storage-homeostasis-pathway (Bandyopadhyay et al., 2022); or direct local AA/metabolite delivery (lysosomal/cytosolic/extracellular-niche), or combination of both, could potentially be helpful to change the course and progression of neurodegenerative-disorders.

## Discussion

### Highlight of future directions

The emerging field of bioengineering-nanotechnology offers enormous potential in treating complex multi-dimensional age-dependent neurodegenerative disorders. The role of cellular-metabolic and quality-control regulatory, lysosome and autophagy-lysosomal-pathway has been unequivocally established in life-threatening neurological disorders. This imposes substantial importance on therapeutically targeting this wing (Bonam et al., 2019). In order to have a successful approach, it is extremely important to know all the intricate details of nanotechnology-biology juncture to produce functional nanoparticles that can be safely delivered in a targeted manner avoiding toxicity. The toxicity-factor is a major obstacle in nanoparticle delivery process, which can be bypassed by thoughtful choice of the nano-materials for manufacturing nanoparticles.

Apart from the non-toxicity feature, consideration of safe delivery mode will be crucial for successful nanoparticle-mediated treatment strategy. Moreover, in the context of autophagy-lysosome-biology, the stability versus biodegradability balance could be a major paradigm that needed to be considered. This can potentially determine the state of lysosomal function and ultimately fate of cellular health. As for example, long-term stability of nanoparticles within the lysosomal lumen can eventually hamper lysosomal function, possibly by altering lysosomal low-pH environment. In fact, similar lysosomal-pH-spoiling effect has been found upon long-term lysosomal gold-nanoparticle accumulation in AD-pathology (Javed et al., 2019). Therefore, careful selection of nanoparticles is crucial to balance their therapeutic benefits versus potential adverse effects. This kind of nanoparticle-mediated lysosomal-poisoning can affect substrate-clearance and consequently perturb lysosomal metabolite storage-homeostasis and ultimately the cellular-metabolic balance. This can be detrimental in case of neurodegeneration, where diseased-neurons are already under severe metabolic stress. In that scenario, designing potentially biodegradable, short-life-spanned, non-toxic nanoparticles would be far more effective in the context of treatment of neurodegenerative disorders.

Another important aspect that requires further discussion is that whether the role of Autophagy manipulation be detrimental or beneficial in the context of neurodegeneration? Being the double-edged sword, any generalized mode of ALP alteration could be tricky and the modulation of the catabolic-pathway would rather have to be case specific. In general, the notion in the field of neurodegeneration is that the upregulation of ALP could be potentially advantageous. However, uncontrolled hyper-activation of this degradative-machinery could have undesired over-catabolic side-effects, which can potentially result in the collapse of the healthy cellular-metabolic environment. Already, in ALS, effectiveness of the hyperactivation of autophagy-lysosomal-wing in improving motor neuronal health is vastly questionable (Bandyopadhyay et al., 2014). Varying choice of the directionality of ALP-regulation in case of particular neurodegenerative disorder would be critical. Although the upregulation of lysosomal-activity upon nanoparticle-mediated lysosomal cathepsin delivery is undeniable, recent studies have identified CathepsinB as a negative regulator of lysosomal calcium-channel, TRPML1. This channel is the upstream regulator/activator of TFEB-activity, that controls autophagy-lysosomal-biogenesis genes, needed for ALP function (Qi et al., 2016). These kinds of dual players have to be considered with care while designing the nano-bioengineered therapeutics.

Thus, the educated selection of targeted intervention avenues, the treatment-delivery mode through bioengineering-nanotechnological advances would be crucial and should be the future focus for successful treatment purposes to better tackle the neurodegenerative and neurological diseases.

## Key concepts

### Tackling neurodegeneration with bioengineering-nanotechnologies: novel prospective study

The hallmark of neurodegeneration is aggregate formation at the cellular-cytosolic-lysosomal level in specific neuronal-population within a pool of healthy-abnormal neuronal-cluster. Applying Nano-Bioengineering driven theranostics - targeted aggregate detection, followed by ‘Autophagy-Lysosomal Pathway’-mediated engulfment-removal, could be an effective treatment-strategy for neurodegeneration.

### Lysosome-autophagy pathway (ALP) modulation: targeting autophagy pathway

The ‘ALP’ consists of upstream cargo-capture and downstream lysosomal-degradation of the captured substrates. Combinatorial genetic and drug targeted modulation of this multi-step clearance pathway consist of autophagosome formation and cargo delivery processes, by using nano-bioengineering devices, could potentially be an effective strategy to better tackle progressive neurodegenerative disorders.

### Lysosome-autophagy pathway (ALP) modulation: targeting directly the organelle - lysosome

For the treatment of neurological-disorder, the contribution of the lysosome, the terminal point of ALP, is vital. Lysosomal alteration through nanotechnology, especially by generating bioengineered super-functional lysosomes with higher aggregate removal ability, by nanoparticle-mediated delivery of activated lysosome-residing enzymes, could be beneficial to arrest neurodegenerative disorders.

### Targeting lysosomes in a *‘Macrophagic Way’* using nano-bioengineering

Smooth targeted delivery of drugs, AA/metabolites, peptides, hormones, and genetic materials (siRNA/shRNA) to the cellular/endo-lysosomal systems of the affected neuronal-population in case of neurodegeneration, can be ensured by bio-engineered macrophages with hyperactivated engulfment-endocytotic-exocytotic property, caused by immuno-inflammatory response triggered in this degenerative disorder.

### Regulation of cellular-lysosomal metabolism regulation by targeted AA/metabolite delivery using nanotechnology-bioengineering in disease

Alteration of niche AAs in treating neurodegeneration are gaining popularity. However, therapeutically, targeted local AA perturbation is far more effective than global change. Therefore, targeted delivery of AA/lysosome-modifiers to alter canonical/noncanonical metabolic pathways, through bioengineered nanoparticle carriers, in affected neurons in neurodegeneration will be highly beneficial.

## Author contributions

AR: Writing – original draft. UB: Conceptualization, Funding acquisition, Investigation, Supervision, Writing – original draft, Writing – review & editing.

## References

[ref1] Abu-Remaileh M. Wyant G. A. Kim C. Laqtom N. N. Abbasi M. Chan S. H.. (2017). Lysosomal metabolomics reveals V-ATPase- and mTOR-dependent regulation of amino acid efflux from lysosomes. Science 358, 807–813. doi: 10.1126/science.aan6298, PMID: 29074583 PMC5704967

[ref2] Alvarez-Erviti L. Seow Y. Yin H. F. Betts C. Lakhal S. Wood M. J. A. (2011). Delivery of siRNA to the mouse brain by systemic injection of targeted exosomes. Nat. Biotechnol. 29, 341–345. doi: 10.1038/nbt.1807, PMID: 21423189

[ref3] Ates B. Koytepe S. Ulu A. Gurses C. Thakur V. K. (2020). Chemistry, structures, and advanced applications of nanocomposites from biorenewable resources. Chem. Rev. 120, 9304–9362. doi: 10.1021/acs.chemrev.9b00553, PMID: 32786427

[ref4] Bandyopadhyay U. Nagy M. Fenton W. A. Horwich A. L. (2014). Absence of lipofuscin in motor neurons of SOD1-linked ALS mice. Proc. Natl. Acad. Sci. U. S. A. 111, 11055–11060. doi: 10.1073/pnas.1409314111, PMID: 25024188 PMC4121794

[ref5] Bandyopadhyay U. Todorova P. Pavlova N. N. Tada Y. Thompson C. B. Finley L. W. S.. (2022). Leucine retention in lysosomes is regulated by starvation. Proc. Natl. Acad. Sci. U. S. A. 119:e2114912119. doi: 10.1073/pnas.2114912119 35105808 PMC8833167

[ref6] Bastaki S. Aravindhan S. Ahmadpour Saheb N. Afsari Kashani M. Evgenievich Dorofeev A. Karoon Kiani F.. (2021). Codelivery of STAT3 and PD-L1 siRNA by hyaluronate-TAT trimethyl/thiolated chitosan nanoparticles suppresses cancer progression in tumor-bearing mice. Life Sci. 266:118847. doi: 10.1016/j.lfs.2020.118847, PMID: 33309720

[ref7] Bento C. F. Renna M. Ghislat G. Puri C. Ashkenazi A. Vicinanza M.. (2016). Mammalian autophagy: how does it work? Annu. Rev. Biochem. 85, 685–713. doi: 10.1146/annurev-biochem-060815-014556 26865532

[ref8] Bonam S. R. Wang F. Muller S. (2019). Lysosomes as a therapeutic target. Nat. Rev. Drug Discov. 18, 923–948. doi: 10.1038/s41573-019-0036-1, PMID: 31477883 PMC7097195

[ref9] Cheng K. K. Chan P. S. Fan S. Kwan S. M. Yeung K. L. Wáng Y. X. J.. (2015). Curcumin-conjugated magnetic nanoparticles for detecting amyloid plaques in Alzheimer's disease mice using magnetic resonance imaging (MRI). Biomaterials 44, 155–172. doi: 10.1016/j.biomaterials.2014.12.005, PMID: 25617135

[ref10] Colacurcio D. J. Nixon R. A. (2016). Disorders of lysosomal acidification-the emerging role of v-ATPase in aging and neurodegenerative disease. Ageing Res. Rev. 32, 75–88. doi: 10.1016/j.arr.2016.05.004, PMID: 27197071 PMC5112157

[ref11] Demeritte T. Viraka Nellore B. P. Kanchanapally R. Sinha S. S. Pramanik A. Chavva S. R.. (2015). Hybrid graphene oxide based Plasmonic-magnetic multifunctional Nanoplatform for selective separation and label-free identification of Alzheimer's disease biomarkers. ACS Appl. Mater. Interfaces 7, 13693–13700. doi: 10.1021/acsami.5b03619, PMID: 26027901 PMC4677996

[ref12] Didiot M. C. Hall L. M. Coles A. H. Haraszti R. A. Godinho B. M. D. C. Chase K.. (2016). Exosome-mediated delivery of Hydrophobically modified siRNA for huntingtin mRNA silencing. Mol. Ther. 24, 1836–1847. doi: 10.1038/mt.2016.126, PMID: 27506293 PMC5112038

[ref13] Duan L. Li X. Ji R. Hao Z. Kong M. Wen X.. (2023). Nanoparticle-based drug delivery systems: an inspiring therapeutic strategy for neurodegenerative diseases. Polymers (Basel) 15:2196. doi: 10.3390/polym15092196, PMID: 37177342 PMC10181407

[ref14] Hajipour M. J. Santoso M. R. Rezaee F. Aghaverdi H. Mahmoudi M. Perry G. (2017). Advances in Alzheimer's diagnosis and therapy: the implications of nanotechnology. Trends Biotechnol. 35, 937–953. doi: 10.1016/j.tibtech.2017.06.002, PMID: 28666544

[ref15] Haney M. J. Zhao Y. Fay J. Duhyeong H. Wang M. Wang H.. (2020). Genetically modified macrophages accomplish targeted gene delivery to the inflamed brain in transgenic Parkin Q311X(a) mice: importance of administration routes. Sci. Rep. 10:11818. doi: 10.1038/s41598-020-68874-7, PMID: 32678262 PMC7366622

[ref16] He C. Klionsky D. J. (2009). Regulation mechanisms and signaling pathways of autophagy. Annu. Rev. Genet. 43, 67–93. doi: 10.1146/annurev-genet-102808-114910, PMID: 19653858 PMC2831538

[ref17] Huang X. Liu C. Kong N. Xiao Y. Yurdagul A. Jr. Tabas I.. (2022). Synthesis of siRNA nanoparticles to silence plaque-destabilizing gene in atherosclerotic lesional macrophages. Nat. Protoc. 17, 748–780. doi: 10.1038/s41596-021-00665-4, PMID: 35121853 PMC9734002

[ref18] Izco M. Blesa J. Schleef M. Schmeer M. Porcari R. al-Shawi R.. (2019). Systemic Exosomal delivery of shRNA Minicircles prevents parkinsonian pathology. Mol. Ther. 27, 2111–2122. doi: 10.1016/j.ymthe.2019.08.010, PMID: 31501034 PMC6904801

[ref19] Jahangard Y. Monfared H. Moradi A. Zare M. Mirnajafi-Zadeh J. Mowla S. J. (2020). Therapeutic effects of transplanted exosomes containing miR-29b to a rat model of Alzheimer's disease. Front. Neurosci. 14:564. doi: 10.3389/fnins.2020.00564, PMID: 32625049 PMC7314926

[ref20] Javed I. Peng G. Xing Y. Yu T. Zhao M. Kakinen A.. (2019). Inhibition of amyloid beta toxicity in zebrafish with a chaperone-gold nanoparticle dual strategy. Nat. Commun. 10:3780. doi: 10.1038/s41467-019-11762-0, PMID: 31439844 PMC6706415

[ref21] Kang Y. J. Cutler E. G. Cho H. (2018). Therapeutic nanoplatforms and delivery strategies for neurological disorders. Nano Converg 5:35. doi: 10.1186/s40580-018-0168-8, PMID: 30499047 PMC6265354

[ref22] Karran E. De Strooper B. (2022). The amyloid hypothesis in Alzheimer disease: new insights from new therapeutics. Nat. Rev. Drug Discov. 21, 306–318. doi: 10.1038/s41573-022-00391-w, PMID: 35177833

[ref23] Kouyoumdjian H. Zhu D. C. el-Dakdouki M. H. Lorenz K. Chen J. Li W.. (2013). Glyconanoparticle aided detection of beta-amyloid by magnetic resonance imaging and attenuation of beta-amyloid induced cytotoxicity. ACS Chem. Neurosci. 4, 575–584. doi: 10.1021/cn3002015, PMID: 23590250 PMC3629742

[ref24] Li T. Yin L. Kang X. Xue W. Wang N. Zhang J.. (2022). TFEB acetylation promotes lysosome biogenesis and ameliorates Alzheimer's disease-relevant phenotypes in mice. J. Biol. Chem. 298:102649. doi: 10.1016/j.jbc.2022.102649, PMID: 36441024 PMC9694136

[ref25] Lin Y. X. Gao Y. J. Wang Y. Qiao Z. Y. Fan G. Qiao S. L.. (2015). pH-sensitive polymeric nanoparticles with gold(I) compound payloads synergistically induce Cancer cell death through modulation of autophagy. Mol. Pharm. 12, 2869–2878. doi: 10.1021/acs.molpharmaceut.5b00060, PMID: 26101892

[ref26] Liu L. Li Y. Peng H. Liu R. Ji W. Shi Z.. (2020). Targeted exosome coating gene-chem nanocomplex as "nanoscavenger" for clearing alpha-synuclein and immune activation of Parkinson's disease. Sci. Adv. 6:eaba3967. doi: 10.1126/sciadv.aba3967, PMID: 33310840 PMC7732192

[ref27] Lu M. Zhao X. Xing H. Liu H. Lang L. Yang T.. (2019). Cell-free synthesis of connexin 43-integrated exosome-mimetic nanoparticles for siRNA delivery. Acta Biomater. 96, 517–536. doi: 10.1016/j.actbio.2019.07.006, PMID: 31284098

[ref28] Maiese K. (2016). Targeting molecules to medicine with mTOR, autophagy and neurodegenerative disorders. Br. J. Clin. Pharmacol. 82, 1245–1266. doi: 10.1111/bcp.12804, PMID: 26469771 PMC5061806

[ref29] Moorthy H. Govindaraju T. (2021). Dendrimer architectonics to treat Cancer and neurodegenerative diseases with implications in Theranostics and personalized medicine. ACS Appl Bio Mater 4, 1115–1139. doi: 10.1021/acsabm.0c01319, PMID: 35014470

[ref30] Neely A. Perry C. Varisli B. Singh A. K. Arbneshi T. Senapati D.. (2009). Ultrasensitive and highly selective detection of Alzheimer's disease biomarker using two-photon Rayleigh scattering properties of gold nanoparticle. ACS Nano 3, 2834–2840. doi: 10.1021/nn900813b, PMID: 19691350 PMC2749966

[ref31] Paskeh M. D. A. Entezari M. Clark C. Zabolian A. Ranjbar E. Farahani M. V.. (2022). Targeted regulation of autophagy using nanoparticles: new insight into cancer therapy. Biochim. Biophys. Acta Mol. basis Dis. 1868:166326. doi: 10.1016/j.bbadis.2021.166326, PMID: 34942307

[ref32] Petcherski A. Tingley B. M. Martin A. Adams S. Brownstein A. J. Steinberg R. A.. (2023). Endo-lysosome-targeted nanoparticle delivery of antiviral therapy for coronavirus infections. bioRxiv. doi: 10.1101/2023.05.08.539898 PMC11790838 39900438

[ref33] Qi X. Man S. M. Malireddi R. K. S. Karki R. Lupfer C. Gurung P.. (2016). Cathepsin B modulates lysosomal biogenesis and host defense against *Francisella novicida* infection. J. Exp. Med. 213, 2081–2097. doi: 10.1084/jem.20151938, PMID: 27551156 PMC5030800

[ref34] Sanchez-Ramos J. Song S. Kong X. Foroutan P. Martinez G. Dominguez-Viqueria W.. (2018). Chitosan-Mangafodipir nanoparticles designed for intranasal delivery of siRNA and DNA to brain. J Drug Deliv Sci Technol 43, 453–460. doi: 10.1016/j.jddst.2017.11.013, PMID: 29805475 PMC5967853

[ref35] Sato H. Takado Y. Toyoda S. Tsukamoto-Yasui M. Minatohara K. Takuwa H.. (2021). Neurodegenerative processes accelerated by protein malnutrition and decelerated by essential amino acids in a tauopathy mouse model. Sci. Adv. 7:eabd5046. doi: 10.1126/sciadv.abd5046 34678069 PMC8535828

[ref36] Siafaka P. I. Okur M. E. Erim P. D. Çağlar E. Ş. Özgenç E. Gündoğdu E.. (2022). Protein and gene delivery Systems for Neurodegenerative Disorders: where do we stand today? Pharmaceutics 14:2425. doi: 10.3390/pharmaceutics14112425, PMID: 36365243 PMC9698227

[ref37] Sillerud L. O. Solberg N. O. Chamberlain R. Orlando R. A. Heidrich J. E. Brown D. C.. (2013). SPION-enhanced magnetic resonance imaging of Alzheimer's disease plaques in AbetaPP/PS-1 transgenic mouse brain. J. Alzheimers Dis. 34, 349–365. doi: 10.3233/JAD-121171, PMID: 23229079 PMC4801216

[ref38] Song W. Soo Lee S. Savini M. Popp L. Colvin V. L. Segatori L. (2014). Ceria nanoparticles stabilized by organic surface coatings activate the lysosome-autophagy system and enhance autophagic clearance. ACS Nano 8, 10328–10342. doi: 10.1021/nn505073u, PMID: 25315655

[ref39] van Sadelhoff J. H. J. Perez Pardo P. Wu J. Garssen J. van Bergenhenegouwen J. Hogenkamp A.. (2019). The gut-immune-brain Axis in autism Spectrum disorders; a focus on amino acids. Front Endocrinol (Lausanne) 10:247. doi: 10.3389/fendo.2019.00247, PMID: 31057483 PMC6477881

[ref40] Wang P. Wu B. Li M. Song Y. Chen C. Feng G.. (2023). Lysosome-targeting aggregation-induced emission nanoparticle enables adoptive macrophage transfer-based precise therapy of bacterial infections. ACS Nano 17, 10365–10375. doi: 10.1021/acsnano.3c00796, PMID: 37235750

[ref41] Weavers H. Evans I. R. Martin P. Wood W. (2016). Corpse engulfment generates a molecular memory that primes the macrophage inflammatory response. Cells 165, 1658–1671. doi: 10.1016/j.cell.2016.04.049, PMID: 27212238 PMC4912690

[ref42] Wong E. Cuervo A. M. (2010). Autophagy gone awry in neurodegenerative diseases. Nat. Neurosci. 13, 805–811. doi: 10.1038/nn.2575, PMID: 20581817 PMC4038747

[ref43] Wu J. de Theije C. G. M. da Silva S. L. Abbring S. van der Horst H. Broersen L. M.. (2017). Dietary interventions that reduce mTOR activity rescue autistic-like behavioral deficits in mice. Brain Behav. Immun. 59, 273–287. doi: 10.1016/j.bbi.2016.09.016, PMID: 27640900

[ref44] Xie Y. X. Naseri N. N. Fels J. Kharel P. Na Y. Lane D.. (2022). Lysosomal exocytosis releases pathogenic alpha-synuclein species from neurons in synucleinopathy models. Nat. Commun. 13:4918. doi: 10.1038/s41467-022-32625-1, PMID: 35995799 PMC9395532

[ref45] Yamamoto H. Zhang S. Mizushima N. (2023). Autophagy genes in biology and disease. Nat. Rev. Genet. 24, 382–400. doi: 10.1038/s41576-022-00562-w, PMID: 36635405 PMC9838376

[ref46] Yoon H. Y. Chang I. H. Goo Y. T. Kim C. H. Kang T. H. Kim S. Y.. (2019). Intravesical delivery of rapamycin via folate-modified liposomes dispersed in thermo-reversible hydrogel. Int. J. Nanomedicine 14, 6249–6268. doi: 10.2147/IJN.S216432, PMID: 31496684 PMC6689153

[ref47] Zhai L. Shen H. Sheng Y. Guan Q. (2021). ADMSC Exo-MicroRNA-22 improve neurological function and neuroinflammation in mice with Alzheimer's disease. J. Cell. Mol. Med. 25, 7513–7523. doi: 10.1111/jcmm.16787, PMID: 34250722 PMC8335682

[ref48] Zheng Y. Su C. Zhao L. Shi Y. (2017). Chitosan nanoparticle-mediated co-delivery of shAtg-5 and gefitinib synergistically promoted the efficacy of chemotherapeutics through the modulation of autophagy. J Nanobiotechnology 15:28. doi: 10.1186/s12951-017-0261-x, PMID: 28399862 PMC5387274

